# Detection and Length Measurement of Cracks Captured in Low Definitions Using Convolutional Neural Networks

**DOI:** 10.3390/s23083990

**Published:** 2023-04-14

**Authors:** Jin-Young Kim, Man-Woo Park, Nhut Truong Huynh, Changsu Shim, Jong-Woong Park

**Affiliations:** 1 Sambo Engineering, Seoul 05640, Republic of Korea; 2 Department of Civil and Environmental Engineering, Myongji University, Yongin 17058, Republic of Korea; 3 Department of Civil and Environmental Engineering, Chung-Ang University, Seoul 06974, Republic of Korea

**Keywords:** deep learning, concrete crack, convolutional neural network, low definition crack image, length measurement

## Abstract

Continuous efforts were made in detecting cracks in images. Varied CNN models were developed and tested for detecting or segmenting crack regions. However, most datasets used in previous works contained clearly distinctive crack images. No previous methods were validated on blurry cracks captured in low definitions. Therefore, this paper presented a framework of detecting the regions of blurred, indistinct concrete cracks. The framework divides an image into small square patches which are classified into crack or non-crack. Well-known CNN models were employed for the classification and compared with each other with experimental tests. This paper also elaborated on critical factors—the patch size and the way of labeling patches—which had considerable influences on the training performance. Furthermore, a series of post-processes for measuring crack lengths were introduced. The proposed framework was tested on the images of bridge decks containing blurred thin cracks and showed reliable performance comparable to practitioners.

## 1. Introduction

In developed countries, as the number of infrastructures aged over 30 years drastically increases, the infrastructure maintenance became a critical issue and drew growing interest from researchers and practitioners. Continuous efforts on effective health monitoring system, as well as recent advances in sensing technology, specialized pieces of equipment, facilitated recording various aspects and states of structural health. For example, varied sensors are deployed on large-scale bridges and tunnels, and the sensing data are accumulated and analyzed for diagnosing the structures. However, despite the potentials of the advanced structural health monitoring (SHM) system, most of the practical tasks still remain manual. Regular inspections always involve visual inspections which require inspectors investigating defects on structures with naked eyes and recording their states. The on-site visual inspection tasks are followed by documentation of the records, including the visualization of the defects on drawings and generating tables of the defect states.

To automate the manual processes, vision-based methods that extract defect information from images or videos were developed. Collecting images or videos is relatively easy and cheap, and the advent of UAV (unmanned aerial vehicle) eases even further, enabling the inspection of the hard-to-reach spots. Given that a vast amount of image or video data are available from recording with mobile phones to scanning with UAVs, image processing and computer vision technologies can increase the level of automation providing contextual information. For this purpose, various measures ranging from image filtering [1,2,3,4,5] to machine vision [6,7,8,9,10,11,12,13,14,15,16,17,18,19,20,21,22,23,24,25,26,27,28,29] were proposed to detect defects on surfaces. From various defect types, a crack is one of the most common and critical defects on concrete elements, and it was targeted to detect and monitor in many research works [1,2,3,4,5,6,7,8,9,10,11,12,13,14,15,16,17,18,19,20,21,22,23,24,25,26,27,28,29,30,31]. Different from general object types such as pedestrian, human face, vehicle, and animals, a crack has no typical shape or appearance patterns that object-detection algorithms can take advantage of. The number of branches, and their length and width can be diverse. Accordingly, it is hard to define and label crack objects, making crack detection challenging.

Initial attempts to detect cracks in images relied mainly on fundamental image features, such as edges [1,2,3,4,5]. However, the processes of retrieving edge features are highly dependent on varied factors—illumination condition, concrete surface colors, shadows, and the level of noises. In other words, no edge-based approach could be generalized to different scenes and conditions. Even when machine learning prevailed in object-detection methods, no significant enhancement of crack detection performance was witnessed because its unstructured shape was hardly trainable. This unresolved problem was tackled by introducing CNNs (convolution neural networks) which showed potential in the field of image recognition and made tremendous progress in object detection. From several branches of frameworks, the mainstream, in principle, works by partitioning an image into small patches and classifying the patches to crack and non-crack regions with the help of CNNs [6,7,8,9,10,11,12,13].

Though the CNN-based crack detection frameworks showed great potential in automating visual inspections, most of the works were tested on the images where cracks appeared clear and distinct, which are associated with thick, deep cracks in severe conditions or close-up views. It is certainly favorable for accurate diagnosis to inspect cracks from their initial states when they look like thin scratches since it allows monitoring the progress of the cracks as they become thicker and deeper. In addition, close-up images are not always available depending on camera specifications and the site conditions. Collecting the images in close-up views requires longer lead time to fully scan a structure, and results in a larger number of images. If cracks are detectable in distant views where they may appear blurry and indistinct, it will widen the range of image collection strategies and reduce the required time. The remote detection can also be helpful when using UAVs which have limited flight time depending on the battery charge. Instead of taking close-up images of intact surfaces unnecessarily, detection on distant view images can tell the spots to capture closely.

This paper proposes a framework for detecting the regions of blurred, indistinct concrete cracks, and measuring their lengths. Following the general procedures of previous works, the framework also divides an image into rectangular patches which will be classified into crack and non-crack regions. For the classification, well-known CNN models are introduced, and their performances are compared with each other in detail. More importantly, this paper also visits critical factors—the patch size and the way of labeling patches—which significantly affect the training outcomes, and thoroughly analyze them to suggest the appropriate training procedures. In addition, a series of steps to measure crack lengths from the crack detection results are introduced. The proposed framework was tested on the images of bridge decks containing blurred thin cracks and showed reliable performance comparable to practitioners.

## 2. Related Works

### 2.1. Crack Detection with Simple Image Features

Continuous research efforts were made for detecting cracks on images. Early stage works focused on the image features that can facilitate isolating the pixel regions of the cracks [1,2,3,4,5]. Accordingly, varied filtering approaches were applied for this purpose. For example, Fujita et al. [1] employed a Hessian-based line filter to detect cracks on concrete structures and Salman et al. [4] applied the Gabor filter to detect cracks on pavements. Since each filter had limitations and was sensitive to image quality or recording conditions, the several combinations of image filters and thresholding algorithms were also presented [2,5]. In addition to the detection stage, crack propagations were monitored by tracking feature points extracted from crack regions [31]. Still, it is barely feasible to find predefined filters that can produce consistent performance on a wide range of noise levels, lighting conditions, irregularly patterned background, structure surfaces, and camera setups [32].

### 2.2. Crack Detection as an Image Patch Classification Problems

The aforementioned limitations were tackled with the advanced approaches on the basis of machine learning algorithms. The advent of CNN drastically enhanced the level of detection-performance-realizing practical applications. One of the categories that apply CNNs for crack detection is the use the CNNs for classifying image patches into crack or non-crack regions [6,7,8,9,10,11,12,13]. Input data to the networks are generally small square regions of original images, of which positive samples include crossing crack trails.

Zhang et al. [6] showed that even a shallow-layered network performed significantly better than earlier machine learning algorithms such as SVM and Boosting. Cha [7] also used a similar size of model that worked in a sliding window framework. This framework basically detected the crack-included patches by applying the CNN model to every single square image cropped by a sliding window. Similarly, Protopapadakis et al. [13] implemented their own CNN model with the post-processing modules for segmenting pixel-wise crack regions and reducing noisy results. On the other hand, well-known CNN models such as VGG-16 [33], ResNet [34], and LeNet-5 [35] were also employed and tested on crack patch datasets [8,10,11,12]. For example, Feng et al. [8] trained the ResNet with an active learning framework and Kim et al. [11] modified LeNet-5 to extract more effective features in crack patches. However, most previous works dealt with images of clear distinctive cracks, and were not validated on thin blurred crack images.

### 2.3. Crack Detection as an Image Segmentation Problem

Although the patch classification approaches showed great potential to be applied to practical applications, their outcome was limited to squared patch-wise regions. Pixel-wise crack regions were not available from the classification-based methods. To fill this gap, CNN-based segmentation approaches were proposed [15,16,17,18,19,20,21,22,23,24,25]. The CNN models used in those approaches were based on the U-Net [36] composed of encoder and decoder.

Cheng et al. [19] applied the U-Net for detecting pavement cracks, which was followed by the developments of U-Net variations [15,16,17,18,22,23,24,25]. Dung and Anh [15] employed the VGG-16 model [33] for both encoder and decoder, and applied it to cropped image datasets. Pantoja-Rosero et al. [22] modified the encoder based on the VGG-11 [33], and enhanced the model to preserve the continuity of crack shapes by selecting the optimal loss function. Liu et al. [16] proposed the customized CNN model that also partly takes after the VGG-16. Their model was characterized by aggregated hierarchical features, which made it feasible to detect cracks at various scales. Similarly, Ren et al. [18] implemented multi-scale feature extraction by embedding a spatial pyramid pooling module. Liu and Wang [25] compared several U-Net variations and found the encoder depth was critical to the accuracy.

While the network models in most works were trained and tested with a certain size of cropped images, Kang and Cha [17] tested their own model on cluttered scenes. Their model included the squeeze and extension-based attention module and worked in a real-time manner. Experimental results showed that cracks in complex scenes can be detected well enough by properly selecting the loss function and the activation functions. However, as mentioned about the classification-based approaches in Section 2.2, segmentation-based approaches were also tested mainly on clear distinctive crack images.

### 2.4. Crack Detection as an Object Detection Problem

The CNN-based object-detection algorithms were also applied for crack detection [26,27,28,29]. Kalfarisi et al. [28] used the faster region-based CNN (Faster R-CNN) [37] and Park et al. [27] used the YOLOv3 (You Only Look Once) [38]. Furthermore, the Mask R-CNN [39] was applied to crack images, adding pixel-wise results onto the detected crack areas [28,40]. Yang et al. [29] presented a comparable framework, but with infrared thermal images. However, despite the performances reported in the previous works, object detection results were limited to the rectangles fitted to cracks appeared in an image. Therefore, the object-detection algorithms were not capable of providing detailed shape information. More importantly, the rectangles were, in general, much larger than the patch sizes used in classification-based methods. Additionally, the cracks detected by the object detection algorithms were mostly unidirectional and had few branches.

## 3. Proposed Framework

As described in the previous section, most works of crack detection were evaluated with clear, distinct crack images. Additionally, the quantification of the detected cracks was not investigated well enough. This paper, defining crack detection as a classification problem, tests well-known CNN models on blurry low-definition cracks with thorough analysis of the factors critical to the training performance. This paper also presents the transition from the patch classification to crack lines as well as their length measurement.

**Figure 1:**
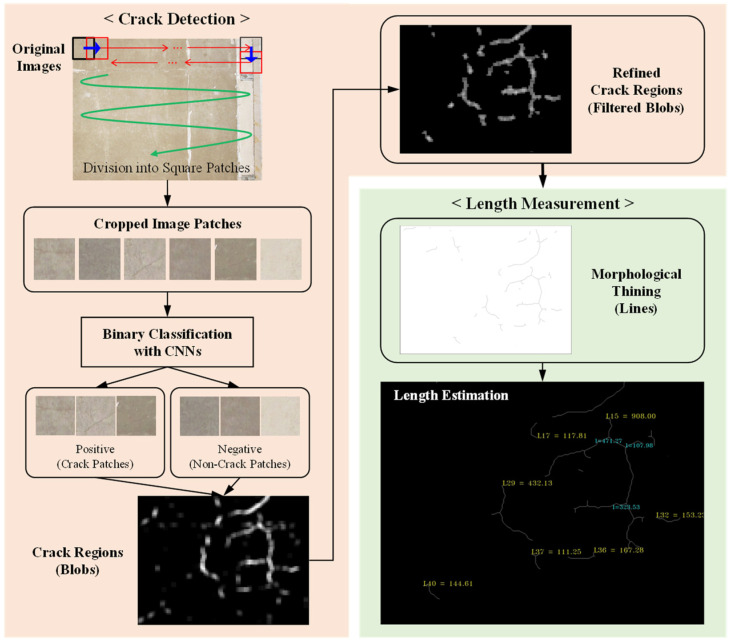
Proposed framework of the crack detection and length measurement.

Figure 1 shows the overall framework that this paper proposes for detecting cracks and measuring their lengths. The original input image is cropped into small square patches in a way that the adjacent patches overlap each other to some extent. The cropped patches are classified as crack or non-crack regions by trained CNNs. The classification results are reflected in a blob image, where a blob represents the region of a connected crack. Once the detection stage is complete, the blobs with low confidence are removed, and the refined blobs are converted to lines. Finally, the lengths of the lines are measured in pixels, and then converted to metric units.

**Figure 2:**
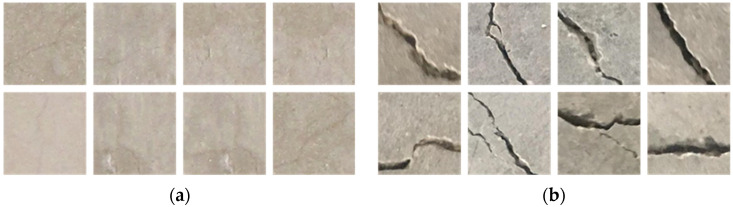
Crack patch samples of (**a**) the dataset used in this paper and (**b**) the dataset from [41].

Despite the efficiency of the segmentation-based approaches which provide geometrical features directly, they were considered unsuitable to the dataset used in this paper. The segmentation-based approaches require labeling crack regions pixel-wisely. As shown in Figure 2a, it was difficult, when compared to Figure 2b, to accurately mark the blurry pixel regions pixel-wisely even with human eyes. In other words, it was barely possible to correctly label the crack regions in pixel-level accuracy. Given that the widths of the cracks in the dataset ranged from one to two pixels, the one-pixel shift of the crack labeling can even have a critical influence on the training performance. On the other hand, the path-wise classification approach was not as sensitive to the labeling as the segmentation-based ones. Accordingly, this paper considered the patch-wise classification preferable to the given dataset.

### 3.1. Transfer Learning of CNN Models

For classifying square patches, several well-known CNN models were employed and compared with each other. Several CNNs with a small number of layers, which were reported to work well for detecting plain cracks, were found inappropriate for low-definition cracks, which will be shown in Section 4. Therefore, this paper opted for noted models—AlexNet [42], VGG-16 [33], and ResNet-152 [34]—which were pre-trained on the ImageNet dataset. Table 1 summarizes the compositions of the three networks. More details can be found in the corresponding research articles [33,34,42].

While the convolutional layers are kept in their original forms, slight modifications are made in the fully connected layers. The convolutional layers of each model are linked to two fully connected layers, of which the number of nodes differs depending on the models and the input patch sizes. The fully connected layers are newly initialized and the ReLU (rectified linear unit) is used for the activation function. The output layer is also changed to contain two nodes corresponding to the binary classes. To embark upon, the front convolutional layers are frozen to retain the pre-trained features, and the fully connected layers are allowed to be trained. Once this stage is complete, the convolutional layers are unfrozen, and the entire network is trained.

**Figure 3:**
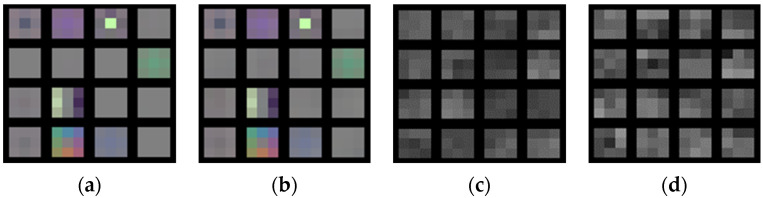
Sample filters of the VGG-16: (**a**) pretrained 1st convolutional layer, (**b**) 1st convolutional layer trained through transfer learning, (**c**) pretrained 10th convolutional layer, (**d**) 10th convolutional layer trained through transfer learning.

The crack shapes and the characteristics of the patch images are radically different from the general images used for pretraining the CNN models. From this perspective, it may not be beneficial to start from the pretrained models. Especially, high-level features trained with general images and crack images would not be close to each other. However, pretrained low-level features related to edges, angles, and corners would still be valid for describing cracks, which makes transfer learning helpful in the proposed framework. In Figure 3, it can be seen that the filters of the first convolutional layer (Figure 3a,b) trained by general images and crack images are similar to each other, which is not the case for those of the tenth convolutional layer (Figure 3c,d).

**Figure 4:**
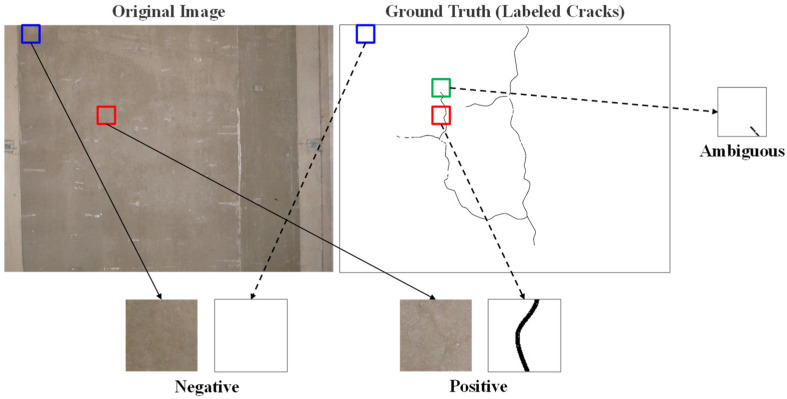
Labeling Cracks on Original Images and Classifying Square Patches.

The performance of machine learning models including CNN is highly dependent on the training dataset. In other words, the square patches cropped from original images need to be labeled accurately based on a consistent standard that conforms to inspectors’ criteria. The standard is crucial for the CNN models to effectively discover the border lines between the classes. The original images are labeled with crack lines, which are confirmed by bridge inspection experts. Accordingly, the original image data are coupled with the ground truth data where cracks are drawn as lines on the white background (Figure 4). Once the coupled images are ready, the original images are divided into a certain size of square patches. As illustrated in Figure 4, during the division, each patch is classified into two classes—crack or non-crack—according to whether its ground truth patch includes crack lines or not. Here, a problem is raised on how to define the inclusion of the crack. It is ambiguous to define the patch that contains a short tail of a crack branch, such as the green patch in Figure 4, as crack or non-crack. It may be advantageous, for example, to classify the patch with a few pixels of cracks as non-crack for CNNs to learn patterns and recognize better the main portions of cracks.

**Figure 5:**
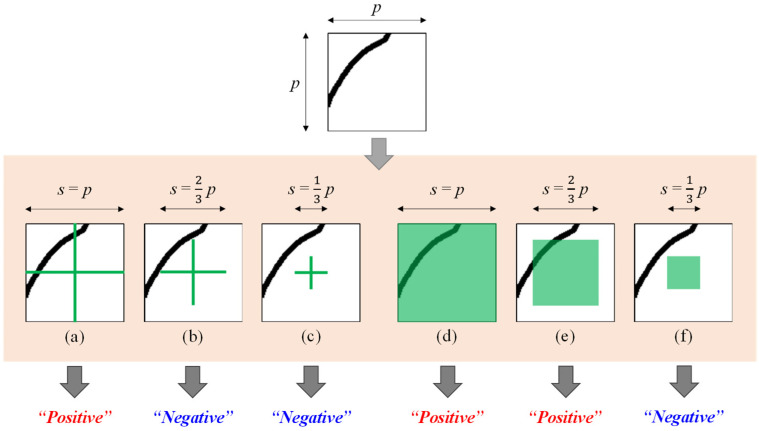
Standards to Define Crack Patches.

Therefore, this paper compares two types of standards to define crack patches. As shown in Figure 5, an image patch is defined as positive (“crack”) if the green cross (Figure 5a–c) or the green square (Figure 5d–f) intersects with cracks. Three sizes of the cross and square were considered, and thus, six standards, in total, were tested. The example sample in Figure 5 would be defined as a crack patch by (a), (d), and (e), while the others define it as a non-crack patch. The cross-based standard is more conservative in defining crack patches and, in general, results in a smaller positive dataset. For instance, the standard (a) in Figure 5 prevents the positive dataset from having a patch which appears intact despite a trace of cracks on it. Similarly, the tighter the cross or square is, the fewer positive patches are generated.

### 3.2. Crack Detection by Classifying Image Patches

**Figure 6:**
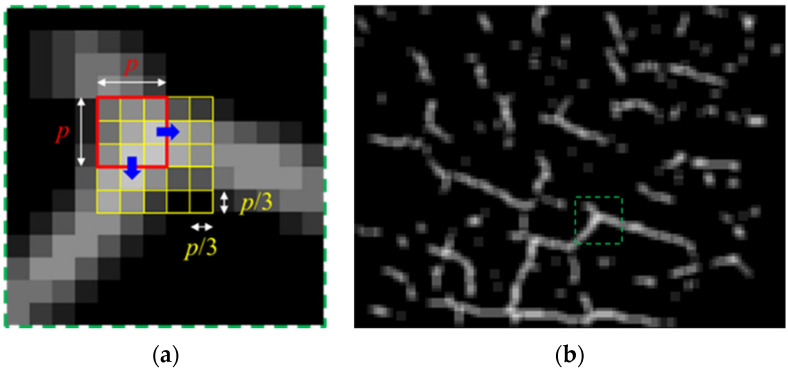
(**a**) Stride of a square region for cropping images; (**b**) crack detection result.

As explained in Figure 1, the trained CNNs are applied to square image patches. The patches are generated by sliding the square across the original images. Figure 6a is the enlarged image of the region marked with a green box in Figure 6b. As shown in Figure 6a, the square strides in a horizontal or a vertical direction by one third of the patch size. Therefore, every pixel is present in nine patches and, accordingly, is involved in nine classifications. In the proposed framework, the counts of positive results from the nine classifications are used to score the level of confidence that the pixels are associated with cracks. Figure 6 shows an example of the results visualizing the ten levels (from 0 to 9) of the confidence levels with gray-scale colors. The brighter the regions are, the more probable they include cracks.

**Figure 7:**
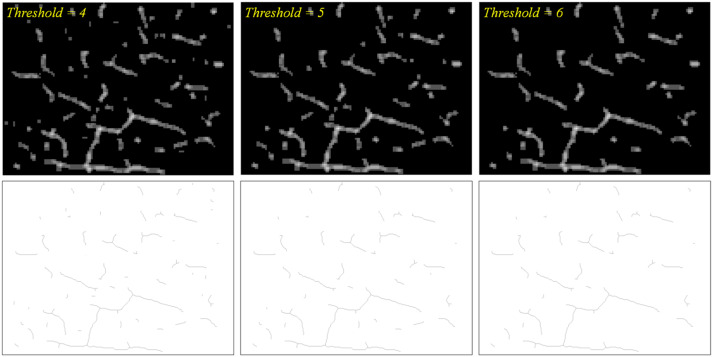
(1st row) Results of filtering pixels and blobs with varied thresholds; (2nd row) results of thinning blobs.

The detection results are further refined by removing the regions of low confidence–dark gray regions. The refinement consists of two steps—the first in a pixel-wise way and the second in a blob-wise way. The first step removes the pixels corresponding to the confidence level lower than 3. Accordingly, the corresponding pixels are changed to black. The second step, considering the connected gray regions as a blob, eliminates the blobs with the maximum confidence lower than a certain threshold. Each blob includes varied confidence levels of regions, and the regions with the confidence lower than a threshold can still be retained if any region in the same blob mark a higher confidence. The first row of Figure 7 shows the refinement of Figure 6b with varied thresholds. It can be observed that the higher threshold clears more of small blobs but is prone to losing actual cracks.

### 3.3. Length Estimation

While the crack widths are barely measurable from the images, their length can be estimated based on the detection results. As a first step to estimate the lengths of the cracks, the refined blobs are transformed into one-pixel wide lines. For this purpose, this paper used the morphological-thinning algorithm [43], which progresses by successively rounding off the boundary pixels on the side of north, east, south, and west, in turn. The second row of Figure 7 demonstrates the results of thinning the images in the first row. It can be observed that the thinning process effectively carry out the conversion to lines while keeping the blobs’ original curved shapes.

**Figure 8:**
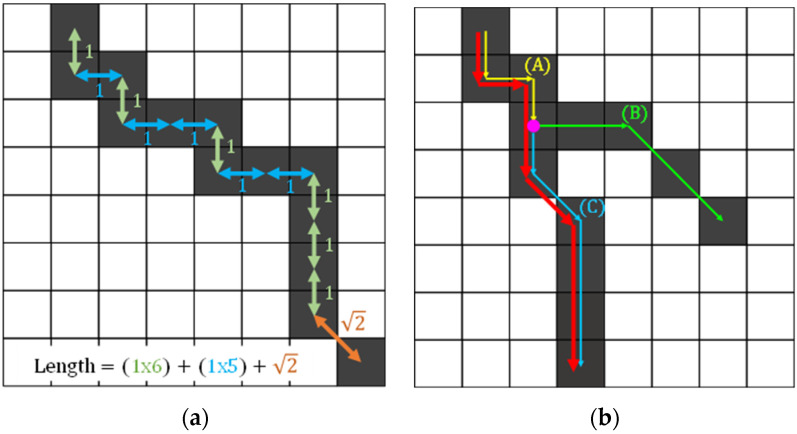
(**a**) Calculation of length segments; (**b**) determination of the main stream and branches.

The length of a crack line extracted from each blob can be measured by tracing the connected pixels of the line from one end to the other end. As shown in Figure 8, the pixels on the line adjoin each other vertically, horizontally, or diagonally. The length segment between two pixels connected in a vertical or horizontal direction is measured as one pixel while those connected diagonally is measured as $\sqrt{2}$ (Figure 8a). Accordingly, by tracing the pixels one by one and accumulating the length segment, the total length of a crack can be directly calculated. For each crack, the pixel with the lowest y coordinate, which corresponds to the top-most point, is chosen for the start of the tracing. During the tracing, black neighbor pixels of the current position, if any, are found and the length segment is calculated based on whether the black pixel is positioned horizontally, vertically, or diagonally.

For the cracks with multiple branches, it is required to measure the lengths of the branches separately. In this research, the branch lengths as well as the total length—the length of the main stream—are calculated simultaneously by a recursive function. The main stream of a crack is defined as the one forming the longest line (the red line in Figure 8b). Therefore, while calculating each branch’s length, the total length can be obtained simply by adding up the length of the longest branch at each confluence. For example, as illustrated in Figure 8b, the length of the branch (A) can be calculated by accumulating length segments starting from the top-most black pixel. When the tracing reaches the confluence where two black neighbors exist, it calculates the lengths of the two branches (B) and (C) and adds up the longer one to the length of the branch (A).

The lengths measured in pixels can be converted to in metric units if the relation between the concrete surface plane and the image plane is known. The original images used in this research are collected with the camera directing perpendicular to the concrete surface. Given the pixel-to-centimeter conversion rate of the images, the crack lengths are finally estimated in centimeters.

## 4. Experiments and Discussions

### 4.1. Dataset

**Figure 9:**
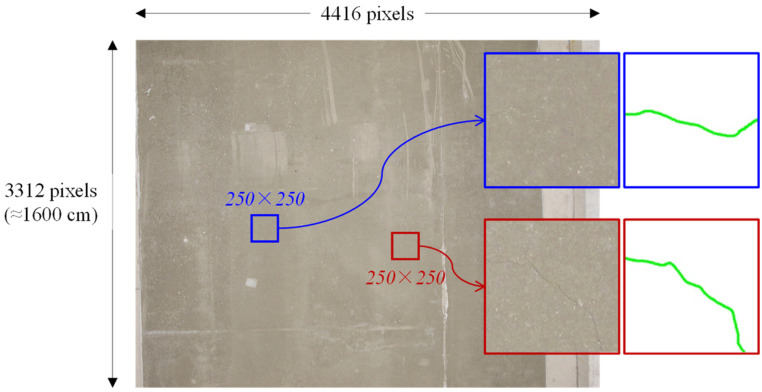
The enlarged views of the 250 × 250 cropped regions containing cracks and the ground truth labeling of the cracks.

The image dataset used in this paper was collected by the bridge monitoring system of Korea Expressway Corporation, which was equipped with cameras that can take images of bridge deck underneath. As mentioned in the previous section, the camera was set up to view the concrete bridge deck perpendicularly from the bottom, and all images were taken at same distance from the surfaces. A total of 134 and 58 images were used for training and test, respectively, and their resolution was 4416 × 3312. Figure 9 shows some examples of the cracks labeled in the original images. The two 250 × 250 regions both contained cracks as labeled with light green line in the corresponding ground truth images. Even in the enlarged views, the cracks were not distinctive, and the one in the blue region was especially barely recognizable. The actual length corresponding to the image height was approximately 1600 cm; thus, the unit conversion rate became 0.483 cm/pixel.

In order to quantify the clarity and sharpness of crack image patches, gradients and pixel differences were calculated. Table 2 shows the comparison with one of the popular crack patch datasets [41] which contained clear thick crack images. A total of 1000 patches from each dataset were randomly selected for the comparison. For each patch, the mean and maximum gradient magnitudes as well as the pixel value range were calculated. In Table 2, the dataset used in this paper exhibited lower scores for all three measures, which confirms that the cracks in the dataset were severely blurred and indistinct when compared to the other dataset.

### 4.2. Evaluation of the Patch Classification

Using the VGG-16 model, two patch sizes and the six standards explained in Section 3.1 and in Figure 5 were tested and evaluated based on precision and recall. In total, 12 cases were compared. The image patches were generated similarly to the way the images are cropped for tests, as explained in Section 3 and in Figure 6a. The only difference was that the negative patches were generated by shifting the sliding window by the patch size (*p*) instead of *p*/3, since negative patches are normally generated more than enough compared to positive patches. The number of training patch data are summarized in Table 3. The smallest number of the positive patches was 23,578 associated with the patch size of 147 and the cross size of 49. For fair comparison, other cases also used the same number of positive patches which were sampled randomly. Negative patches were sampled three times more than the positive for all cases.

**Figure 10:**
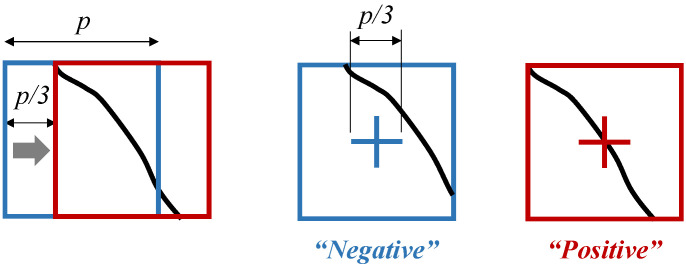
The stride of one third patch size can complement the CNNs trained with a conservative standard.

In the training, 25% of the training data was used for validation. Table 4 shows the precision and the recall evaluated on the validation dataset. Each training was stopped when overfitting was observed, and the model obtained at the iteration with the lowest validation error was selected. Regarding the patch size, 99 × 99 patches were found more appropriate than 147 × 147 patches to isolate crack feature. In terms of the labeling standards, the cross-based standards proved better to separate the crack patches that the CNN could effectively distinguish. Additionally, the smaller size of the cross or the square was used, the more accurately the CNN classified the crack patches. It can be inferred that more conservative standards were preferred by the CNN. However, the conservative standards were prone to miss the patches that marginally intersected a crack. The limitation of a standard itself was not reflected in Table 4 since this evaluation itself was made based on each one’s own standard. This hidden limitation can still be alleviated by the one third stride of the square sliding window. Figure 10 illustrates an example. Even when the CNN could not classify the blue patch as positive, the stride of one third patch size could position the crack on the center in the red patch and provide a higher chance to classify the region as positive.

The patch size of 45 × 45 was excluded in the experiments based on the preliminary experimental results which showed absolutely lower performance. From the experiments, it can be inferred that for blurred cracks which are 1–2 pixels wide, around 99 × 99 would be the appropriate patch size to classify their inclusions. It should be noted that the smaller patch sizes are advantageous when evaluating based on pixel-wise precision and recall as well as when thinning the blobs and retrieving more accurate crack shapes. Regarding the cross or square sizes, though the experiments compare only three values, it can be confirmed that their sizes have to be set smaller than the patch size to achieve optimal performance, and roughly one-third of the patch size is a good starting point.

Using the patch size of 99 and the data labeled by the cross-based standard with the size of 33, three well-known CNN models were compared. The comparison is summarized in Table 5. Other than the AlexNet resulting in low recall, the other three models were found comparable to each other. It can be inferred that the low recall of the AlexNet was attributed to its simple configuration consisting of far fewer layers than other models. Another simple CNN model [7], composed of four convolutional layers, was implemented for comparison. The model included max pooling layers and dropouts. The model was trained with the dataset used in this paper, and the test results exhibited only around 67% of precision and recall. It also signified the limitation of the simple networks on extracting features of indistinct cracks, and the needs for more complex CNN models.

While the AlexNet and the ResNet 152 contained the convolutional layers with the kernel sizes of 11 × 11, 7 × 7, or 5 × 5, the VGG-16 was composed of the convolutional layers with the kernel size of 3 × 3. It signifies that larger kernel sizes do not have a critical impact on the performance of detecting blurred indistinct cracks. The batch size was set to 50 in all experiments. Several attempts with a few variations of the batch sizes were made to find no significant improvements.

### 4.3. Evaluation of the Crack Detection

**Figure 11:**
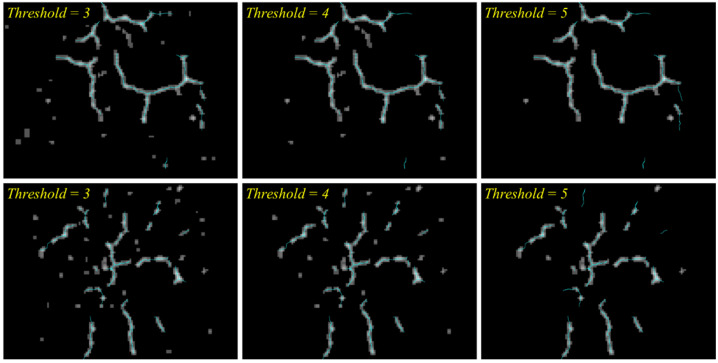
Examples of the blob images visualized with the ground truth crack lines.

Once an optimal CNN model is chosen, the proposed framework can be applied to test images. Giving priority to recall, the VGG-16 was used for testing. As presented in Section 3.2, the patch classification results were integrated and visualized in gray-scale blob images. The blob images were evaluated based on the precision and recall calculated pixel-wise as other segmentation-based methods were generally evaluated. Table 6 shows that the precision grew higher as the threshold for blob refinement increases while the recall behaved in the opposite way. The transition from the threshold of 4 to 5 dropped the recall drastically, indicating that it is optimal to retain the blobs which contain any single pixel classified as positive at least four times from nine classifications. Figure 11 presents the blob images of two test images with varied thresholds, on which the ground truth cracks were drawn with cyan lines.

Pixel-wise Precision = TP/(TP + FP) × 100(1)

Pixel-wise Recall = TP/(TP + FN) × 100(2)

- TP: # of pixels corresponding to crack regions and retrieved as crack regions;
- FP: # of pixels corresponding to non-crack regions but retrieved as crack regions;
- FN: # of pixels corresponding to crack regions but retrieved as non-crack regions.

The pixel-wise precision was significantly low, approximately ranging 2.0–3.0%. However, the low values actually represent a very reasonable performance. The detection results are blobs which are the unions of the square patches, and the pixels on the crack account for only a small portion. Hence, for example, if a single line of crack crosses a 99 × 99 patch horizontally, the pixel-wise precision of the patch region is about 1.0% (=99/(99 × 99)). If the cracks are labeled with 4-pixel wide lines, the precision becomes 4%. Accordingly, the pixel-wise precision is not a proper metric to fairly evaluate the crack detection which works mainly by patch classification. It should be noted that the framework relying on the patch classification focuses on retrieving dilated regions of cracks rather than pinpointing the pixels on the cracks as segmentation-based methods do.

The DeepCrack [16], which is a segmentation-based method, was also implemented for comparison. While the pixel-wise precision was 79.40%, the pixel-wise recall was 1.46%. The recall value indicates that the segmentation-based approaches were not appropriate to the image dataset used in this research. It shows that the methods that perform very well in detecting distinct cracks do not guarantee comparable results when applied to blurred noisy images or initial states of cracks.

### 4.4. Evaluation of the Length Estimation

As described in Section 3.3, the filtered blob regions were converted to lines with the thinning algorithm and the lines lengths were calculated based on the conversion rate of 0.483 cm/pixel. For each crack, the total length which is the summation of the longest branches was calculated and compared with the corresponding measurement records in inspection reports. A total of 45 crack lengths were investigated, and the comparison results in 9.87% error in average. Table 7 summarizes the length estimation errors. It should be noted that the records in the reports also contain errors which may be higher than the errors of the proposed framework, and the comparison was to signify the potential feasibility of the framework replacing the current practices.

Length estimation error = (*l*_estimated_ − *l*_inspected_)/*l*_inspected_ × 100 (3)

- *l*_estimated_: estimated length
- *l*_inspected_: length recorded in inspection reports

## 5. Conclusions

Although continuous efforts were made on automated crack detection, most works were validated on the datasets of cracks that were clearly distinctive and thick enough. This paper explored the image dataset of thin blurry concrete cracks. Based on the thorough reviews of the previous works and preliminary tests, patch-wise classification was chosen as the best method for detecting the indistinctive cracks. To this end, well-known CNNs including AlexNet, VGG-16, and ResNet-50 were trained with certain sizes of patches. The trained CNNs were utilized with a sliding window to detect cracks on original images. Blob filtering and morphological thinning were applied to the CNN outputs, providing crack shapes and length measurements. The strategies of preparing patch datasets and training the models were detailed with experimental results. The results show that it is feasible to detect and measure blurry cracks by using relatively deeper layers of CNNs. Additionally, it was found that the patch-wise classification approaches were more suitable than the CNN-based segmentation methods.

It is expected that the proposed framework and presented results would add more functionalities of vision-based structural inspection and UAV-based monitoring in the future. Future work will include the analysis of the potential positive effect of detecting blurred cracks on UAV-based structural inspection. Additionally, the feasibility of applying the proposed approach in other conditions, such as underwater environments where it is relatively hard to capture clear high-quality images, can also be investigated.

## Figures and Tables

**Table 1:** Summary of the CNN models.

Network Models	AlexNet	VGG-16	ResNet-152
# of convolutional layers	5	13	151
# of parameters in convolutional layers	3,747,200	14,714,688	58,143,810
Kernel sizes	11 × 11, 5 × 5, 3 × 3	3 × 3	7 × 7, 3 × 3, 1 × 1

**Table 2:** Comparison of the crack patch datasets in terms of clarity and sharpness.

Measures		Dataset [43]	Blurred Crack Patches
Gradient Magnitude	Mean	33.49	21.89
	Max.	501.55	210.30
Mean Pixel Value Range *		194.29	84.37

* Pixel Value Range = (maximum pixel value–minimum pixel value).

**Table 3:** The number of generated image patches for training CNNs.

Patch Size * (*p*)	Cross/Square Size * (*s*)	Cross-Based		Square-Based	
		Positive	Negative	Positive	Negative
99	33	37,426	190,351	49,657	188,940
	67	58,321	188,000	80,344	185,570
	99	99,401	183,436	143,253	178,587
147	49	23,578	85,866	32,287	84,853
	99	37,076	84,341	52,626	82,553
	147	64,188	81,199	95,982	77,651

* The values are in pixels.

**Table 4:** Precision and recall of classifying crack patches depending on the patch sizes and labeling standards.

Patch Size * (*p*)	Cross/Square Size* (*s*)	Cross-Based		Square-Based	
		Precision (%)	Recall (%)	Precision (%)	Recall (%)
99	33	88.76	87.14	87.12	72.22
	67	81.08	81.08	59.16	78.38
	99	77.19	77.48	67.41	63.21
147	49	74.55	86.63	75.42	65.02
	99	56.59	86.87	65.57	79.78
	147	64.20	78.98	71.78	63.30

* The values are in pixels.

**Table 5:** Comparison of the well-known CNN models.

CNN	Precision (%)	Recall (%)
AlexNet	87.74	77.77
VGG-16	88.76	87.14
ResNet 152	87.59	85.47

**Table 6:** Pixel-wise precision and recall.

Threshold	Pixel-Wise Precision (%)	Pixel-Wise Recall (%)
No refinement	1.95	97.89
3	2.31	97.63
4	2.48	97.00
5	2.70	64.51
6	2.97	86.94

**Table 7:** Summary of the length estimation errors (%).

Average	Standard Deviation	Minimum	Maximum
9.87	7.20	0.35	23.91

## Data Availability

The data presented in this study are available upon request from the corresponding author.
